# Urinary proteomics in chronic kidney disease: diagnosis and risk of progression beyond albuminuria

**DOI:** 10.1186/s12014-015-9092-7

**Published:** 2015-08-07

**Authors:** Marius A Øvrehus, Petra Zürbig, Bjørn E Vikse, Stein I Hallan

**Affiliations:** Department of Nephrology, St Olav University Hospital, Trondheim, Norway; Department of Cancer Research and Molecular Medicine, Faculty of Medicine, Norwegian University of Science and Technology, Trondheim, Norway; Mosaiques Diagnostics GmbH, Hannover, Germany; Renal Research Group, Department of Clinical Medicine, University of Bergen, Bergen, Norway; Department of Medicine, Haugesund Hospital, Haugesund, Norway; Center of Renal Translational Medicine, University of California San Diego (UCSD), La Jolla, USA

**Keywords:** Chronic kidney disease, Hypertensive nephropathy, Urine, Albuminuria, Proteomics, Disease progression

## Abstract

**Background:**

The contrast between a high prevalence of chronic kidney disease (CKD) and the low incidence of end-stage renal disease highlights the need for new biomarkers of progression beyond albuminuria testing. Urinary proteomics is a promising method, but more studies focusing on progression rate and patients with hypertensive nephropathy are needed.

**Results:**

We analyzed urine samples with capillary electrophoresis coupled to a mass-spectrometer from 18 well characterized patients with CKD stage 4–5 (of whom six with hypertensive nephropathy) and 17 healthy controls. Classification scores based on a previously developed panel of 273 urinary peptides were calculated and compared to urine albumin dipstick results. Urinary proteomics classified CKD with a sensitivity of 0.95 and specificity of 1.00. Overall diagnostic accuracy (area under ROC curve) was 0.98, which was better than for albuminuria (0.85, *p* = 0.02). Results for hypertensive nephropathy were similar to other CKD diagnoses. Adding the proteomic score to an albuminuria model improved detection of rapid kidney function decline (>4 ml/min/1.73 m^2^ per year) substantially: area under ROC curve increased from 0.762 to 0.909 (*p* = 0.042), and 38% of rapid progressors were correctly reclassified to a higher risk and 55% of slow progressors were correctly reclassified to a lower risk category. Reduced excretion of collagen types I–III, uromodulin, and other indicators of interstitial inflammation, fibrosis and tubular dysfunction were associated with CKD diagnosis and rapid progression. Patients with hypertensive nephropathy displayed the same findings as other types of CKD.

**Conclusions:**

Urinary proteomic analyses had a high diagnostic accuracy for CKD, including hypertensive nephropathy, and strongly improved identification of patients with rapid kidney function decline beyond albuminuria testing.

## Background

Chronic kidney disease (CKD) has a high prevalence and represents a large burden of morbidity and health care cost [1, 2]. Diagnosis and staging is based on estimated glomerular filtration rate (eGFR) and the degree of albuminuria, which currently is our most reliable marker of rapid kidney function decline [3]. However, the diagnostic accuracy of albuminuria for CKD is only moderate with most studies reporting area under the ROC curve ranging 0.80–0.85 [4–8], so predicting which CKD patients will have a more rapid disease progression remains a major clinical problem.

Emerging gene-based tests typically report relative risks of 1.2–1.4 for polygenetic diseases like hypertension and CKD, which renders them largely useless as diagnostic tools [9]. Other recent technologies enable us to detect large numbers of proteins and metabolites in urine, and these technologies may have a greater potential as they focus on the end products of biological processes. Several studies have used urine proteomics for diagnostic purposes in glomerulonephritis [10], renal cancer [11] and renal transplantation [12], but there is a strong call for more clinically relevant studies and better phenotyping [13]. Despite making up 30% of ESRD cases in the US and Europe, hypertensive nephropathy is surprisingly understudied, and proteomic analyses has never been performed [14]. The diagnosis of hypertensive nephropathy is based on unspecific clinical characteristics, and the pathophysiology of this broad clinically-based entity is not well described and could be different from what has been described in experimental and biopsy based studies.

Our study describes phenotype characteristics, progression rates, and outcomes in unselected Norwegian CKD outpatients, and relate them to their urine proteomic findings, with special attention to clinically diagnosed hypertensive nephropathy.

## Results

Eighteen CKD stage 4–5 patients with a wide range of kidney diagnoses were included: eight with glomerulonephritis/diabetic nephropathy, six with hypertensive nephropathy and four cases with miscellaneous causes of CKD (lithium nephropathy, Alport disease, chronic interstitial nephritis, and cyclosporine A toxicity). Seventeen healthy controls were also included. Baseline demographics and kidney status are described in Table 1. As expected, diabetes, cardiovascular disease, and kidney related variables were substantially worse in the CKD group, but their levels of blood pressure, hemoglobin, phosphate, parathyroid hormone (PTH) and other markers of uremia indicated that they were reasonably well treated and in an acceptable metabolic state. Mean eGFR at inclusion was 17 ml/min/1.73 m^2^ in the CKD cases, and their mean decline in kidney function over the past 1–11 years prior to inclusion was very similar to the decline over the two and a half years following inclusion [−8.1 ml/min/1.73 m^2^/year (±8.4) vs −8.8 ml/min/1.73 m^2^/year (±7.6), *p* = 0.75, based on a mean of 8.9 and 2.2 measurements per subject]. We therefore used data from the total observation period to indicate their rate of progression. Controls did not show any significant decline in kidney function over the 2 years, and we display baseline characteristics for rapid progressors (eGFR decline >4 ml/min/1.73 m^2 ^per year) versus slow progressor (eGFR decline ≤4 ml/min/1.73 m^2 ^per year). Patients with hypertensive nephropathy typically had higher age, less albuminuria, and slightly slower decline in kidney function compared to patients with glomerulonephritis and diabetes nephropathy. By 2012, three CKD patients were on dialysis, four had been transplanted, and six had died.

**Table 1:** Baseline characteristics of participants

	Major groups		Progression rate		CKD diagnosis		
	Healthy (17)	CKD (18)	Rapid (13)	Slow (22)	HN (6)	GN/DN (8)	Other (4)
Age	47.8 (10.6)	63.7 (16.3)	63.8 (18.3)	51.5 (13.0)	77.8 (4.9)	61.6 (12.3)	47.0 (20.3)
Male gender (%)	58.8	72.2	84.6	54.5	100	87.5	0.0
Diabetes Mellitus (%)	0.0	33.3	38.5	4.5	33.3 *	37.5	0.0
Cardiovascular disease (%)	0.0	55.6	53.8	13.6	83.3	37.5	50.0
eGFR (ml/min/1.73 m^2^)	87.2 (5.4)	17.4 (7.2)	16.6 (6.9)	71.0 (30.2)	16.7 (8.6)	19.6 (7.7)	14.0 (1.4)
eGFR (ml/min/1.73 m^2^) decline per year	−0.3 (1.4)	−6.7 (5.1)	−8.9 (4.6)	−0.6 (1.4)	−5.8 (1.9)	−6.4 (5.1)	−8.8 (8.4)
Albuminuria (dipstick)							
Trace/+ (%)	17.7	22.2	23.1	18.2	33.3	25.0	0.0
++/+++ (%)	0.0	55.6	61.6	9.1	33.3	62.5	75.0
Systolic BP (mmHg)	131.8 (14.3)	144.2 (24.6)	144.1 (28.5)	134.8 (15.5)	142.2 (21.1)	146.6 (32.5)	142.3 (20.0)
Hgb (g/dl)	14.2 (1.3)	11.6 (1.5)	11.5 (1.3)	13.6 (1.7)	11.2 (1.7)	11.9 (1.6)	11.8 (1.1)
K (mmol/l)	4.1 (0.3)	4.5 (0.6)	4.6 (0.6)	4.2 (0.4)	4.6 (0.6)	4.7 (0.5)	4.2 (0.7)
Ca (mmol/l)	2.3 (0.1)	2.3 (0.1)	2.3 (0.2)	2.3 (0.1)	2.3 (0.2)	2.3 (0.1)	2.2 (0.02)
P (mmol/l)	1.1 (0.1)	1.4 (0.5)	1.5 (0.5)	1.1 (0.2)	1.6 (0.7)	1.3 (0.4)	1.4 (0.3)
Urea (mmol/l)	6.1 (1.1)	23.5 (8.5)	24.1 (8.3)	10.0 (8.2)	26.0 (10.7)	22.9 (5.8)	21.3 (10.3)
PTH (pmol/l)	3.5 (0.7)	28.2 (23.4)	30.5 (27.0)	7.2 (10.3)	25.3 (17.4)	29.3 (36.0)	30.0 (15.1)
Bicarbonate (mmol/l)	24.9 (2.4)	20.8 (2.3)	21.0 (2.4)	24.0 (3.0)	20.7 (2.9)	21.0 (2.2)	20.5 (1.9)

Data are mean (1SD) or percentages. Rapid progressors: eGFR declined more than 4 ml/min/1.73m2 per year. Slow progressors: eGFR declined less than 4 ml/min/1.73 m^2^ per year.

*GN* glomerulonephritis, *DN* diabetic nephropathy, *HN* hypertensive nephropathy, *Other* other CKD diagnosis.

* One patient fulfilled the diabetes criteria just before study inclusion and another had nephrosclerosis only in his kidney biopsy.

**Fig. 1:**
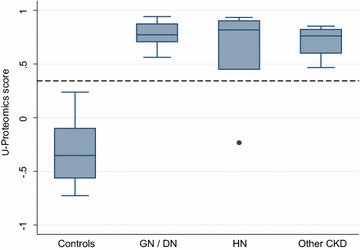
Urine proteomics classification score (CKD273) by CKD diagnosis. GN/DN: patients with glomerulonephritis or diabetes nephropathy; HN: patients with hypertensive nephropathy. Negative values indicate normal healthy subjects, and 0.343 have been used as cut-off for CKD (*dotted line*) [10].

**Fig. 2:**
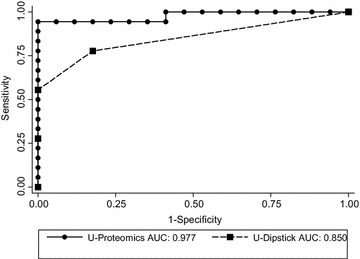
Receiver Operating Characteristics (ROC) analysis of urine proteomics (CKD273 classifier) and albuminuria (dipstick) for diagnosing patients with CKD. *AUC* area under curve.

The urine proteomic analyses detected 4,276 different proteins, and information from 273 of these were converted into a classification score for each subject with values above the predefined 0.343 cutoff indicating high probability for CKD [10]. The mean score in CKD patients and controls were 0.71 and −0.31, respectively (*p* < 0.001), indicating excellent overall discrimination. The box-and-whisker plots in Fig. 1 show the distribution of the proteomics scores by CKD diagnosis. Classification scores were higher than the cut-off value in all CKD patients, except for one patient with hypertensive nephropathy. The proteomics score had a sensitivity of 95% and a specificity of 100% using the standard cut-off of 0.343, and the overall diagnostic accuracy was also excellent [area under ROC curve 0.977 (95% confidence interval (CI) 0.930–1.000)] (Fig. 2). ROC analysis of the urine dipstick test for albuminuria gave an AUC of 0.850 (95% CI 0.730–0.970), which is a significantly lower diagnostic accuracy (*p* = 0.02).

**Fig. 3:**
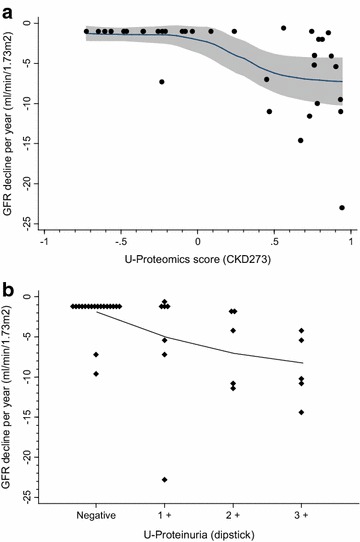
Association between kidney function decline per year (%) and urinary proteomic score (**a**) and dipstick albuminuria (**b**).

Figure 3a shows the continuous relationship between urine proteomics score and kidney function decline. Kidney function deteriorated substantially in the proteomics scores range 0.0–0.5, while the association was rather flat for scores above 0.5 with a decline in eGFR of 7 ml/min/1.73 m^2^ per year. The corresponding relationship for albuminuria is shown in Fig. 3b. Kidney function decline per year increased with higher grades of albuminuria, but the figure also demonstrates substantial variation in kidney function decline within each level of albuminuria. Albuminuria had an area under the ROC curve of 0.762 for detecting subjects with rapid kidney function decline (more than 4 ml/min/1.73 m^2^ per year). Corresponding results for the urine proteomics score was 0.864. Adding proteomic score to an albuminuria model, which is a clinically relevant evaluation, significantly increased the diagnostic accuracy to AUC 0.909 (p = 0.042 compared to albuminuria alone). Furthermore, reclassification analysis showed that two out of five rapid progressors with intermediate predicted risk were (correctly) reclassified into the high risk group, while one out of five was (incorrectly) reclassified to the low risk group. For the slow progressors, three out of four with intermediate risk were (correctly) reclassified into the low risk group (Table 2).

**Table 2:** Risk reclassification when adding urine proteomic score to albuminuria for predicting risk of rapid kidney function decline

	Predicted risk for having rapid kidney function decline			
	0–9%	10–49%	50–100%	Total (%)
Subjects with rapid kidney function decline				
Model with albuminuria	0.0%	38.5%	61.6%	100.0
Model with albuminuria + proteomics	7.7	15.4	76.9	100.0
Subjects without rapid kidney function decline				
Model with albuminuria	0.0	90.9	9.1	100.0
Model with albuminuria + proteomics	68.2	22.7	9.1	100.0

Proteomic intensities of the most important urinary proteins are given in Table 3 for the different diagnostic groups compared to healthy controls. The associations of specific urinary proteins to CKD diagnosis and to rapid kidney function decline are given in Table 4. Data are presented as standardized beta-coefficients, i.e. effect on outcome per 1 SD change to facilitate comparisons, and as area under ROC curves. Fragments from collagen types I, II, and III were strongly reduced and ranked on top with typical ORs of 0.01 (i.e. if collagen concentration decreases with 1 SD the risk increases 100 times) and ROC areas of 0.95 (i.e. the test correctly classifies 95% of pairs with and without the outcome). No association was found to collagen type IV, which is the dominant glomerulus basement membrane type. Urine levels of CD99, uromodulin, sodium/potassium-transporting ATPase gamma chain, and osteopontin were also reduced in CKD patients. Except for osteopontin, these proteins were also strongly associated with a rapid decline in kidney function. The systemic blood-derived proteins were typically lower ranked with ROC area-under-curves below 0.80, and albumin was ranked number 15 for rapid kidney decline.

**Table 3:** Amplitudes of most important urinary protein by clinical diagnosis

Peptide information	SwissProt name	Mean amplitude				Fold changes			
Peptide name		Control	HN	GN + DN	Others	Control	HN	GN + DN	Others
Alpha-1-antitrypsin	A1AT_HUMAN	39	11,031	21,119	21,236	1	282.5	540.9	543.9
Serum albumin	ALBU_HUMAN	0	23,348	78,627	13,663				
Apolipoprotein A-I	APOA1_HUMAN	47	127,871	110,906	38,500	1	2,713.9	2,353.8	817.1
Na/K-transp. ATPase gamma chain	ATNG_HUMAN	1,984	396	322	450	1	0.2	0.2	0.2
Beta-2-microglobulin	B2MG_HUMAN	0	239,173	677,860	207,089				
CD99 antigen	CD99_HUMAN	1,357	0	28	129	1	0.0	0.0	0.1
Collagen alpha-1 (I) chain	CO1A1_HUMAN	3,078	1,533	1,123	2,455	1	0.5	0.4	0.8
Collagen alpha-1 (II) chain	CO2A1_HUMAN	3,190	1,344	500	1,367	1	0.4	0.2	0.4
Collagen alpha-1 (III) chain	CO3A1_HUMAN	2,338	1,064	717	1,246	1	0.5	0.3	0.5
Alpha-2-HS-glycoprotein	FETUA_HUMAN	43	12,128	14,441	6,950	1	283.7	337.8	162.5
Fibrinogen alpha chain	FIBA_HUMAN	965	14,993	2,308	6,477	1	15.5	2.4	6.7
Osteopontin	OSTP_HUMAN	410	0	0	39	1	0.0	0.0	0.1
Membrane associated progesterone receptor component 1	PGRC1_HUMAN	536	1,017	8	502	1	1.9	0.0	0.9
Polymeric-immunoglobulin receptor	PIGR_HUMAN	1,624	583	584	740	1	0.4	0.4	0.5
Transthyretin (Prealbumin)	TTHY_HUMAN	15	22,299	53,034	22,292	1	1,471.6	3,499.9	1,471.2
Uromodulin	UROM_HUMAN	2,525	89	136	176	1	0.0	0.1	0.1
Neurosecretory protein VGF	VGF_HUMAN	1,770	9,796	6,140	5,644	1	5.5	3.5	3.2

*HN* Hypertensive nephropathy, *GN* Glomerulonephritis, *DN* Diabetic nephropathy.

**Table 4:** Specific urinary proteins listed by association and diagnostic accuracy for CKD and rapid kidney function decline

Protein	Detected peptides	CKD diagnosis				Rapid kidney function decline			
		P value	OR (StdX)	ROC	Rank	P value	OR (StdX)	ROC	Rank
Collagen alpha-1 (I) chain	33	0.002	0.03	0.941	1	0.004	0.101	0.853	3
CD99 antigen	1	0.004	0.001	0.918	2	0.028	0.008	0.832	5
Uromodulin	3	0.007	0.001	0.98	3	0.019	0.025	0.846	4
Sodium/potassium-transporting ATPase gamma chain	1	0.002	0.032	0.954	4	0.016	0.002	0.93	2
Collagen alpha-1 (II) chain	1	0.002	0.04	0.948	5	0.001	0.063	0.93	1
Collagen alpha-1 (III) chain	15	0.002	0.112	0.882	6	0.013	0.195	0.837	6
Neurosecretory protein VGF	1	0.008	37	0.876	7	0.017	4.58	0.825	7
Osteopontin	2	0.016	0.018	0.863	8	0.99		0.5	18
Collagen alpha-2 (I) chain	4	0.012	0.172	0.848	9	0.069	0.35	0.724	13
Transthyretin (Prealbumin)	2	0.086	n.s.	0.892	10	0.062	5.51	0.82	11
Beta-2-microglobulin	1	0.065	n.s.	0.859	11	0.358	1.39	0.811	17
Alpha-2-HS-glycoprotein	2	0.05	n.s.	0.84	12	0.07	2.33	0.86	12
Alpha-1-antitrypsin	3	0.168	n.s.	0.871	13	0.047	11.11	0.811	10
Polymeric-immunoglobulin receptor	1	0.02	0.337	0.77	14	0.097	0.48	0.706	16
Apolipoprotein A-I	1	0.172	n.s.	0.85	15	0.047	9.45	0.822	9
Membrane associated progesterone receptor component 1	1	0.056	0.441	0.801	16	0.103	0.429	0.731	14
Albumin	1	0.397	n.s.	0.845	17	0.17	1.77	0.745	15
Fibrinogen alpha chain	2	0.156	n.s.	0.722	18	0.03	9	0.822	8

Protein names and number of specific peptides detected from this protein are given. Data are given only for the best peptide per protein. Odds ratios are based on unadjusted logistic regression analysis. Peptides with *p* values <0.10 or with special interest (albumin) were included. Data show *p* value and odds ratio for outcome associated with one standard deviation change of protein to improve comparability (logistic regression analysis; OR StdX could not be calculated for all associations). Area under the ROC curve is also given. The separate rankings for CKD diagnosis and rapid kidney function decline (>4 ml/min/1.73 m^2^ per year) represent the mean ranks for *p* values, standardized OR and ROC.

*n.s.* not significant.

## Discussion

We found that a urinary proteomics classification score based on 273 different proteins had a significantly better diagnostic accuracy for chronic kidney disease than albuminuria. Adding the proteomic score to albuminuria, our currently best predictor of kidney prognosis, improved detection of patients with rapid progression substantially. Reduced urinary excretion of collagen fragments types I–III was among the most important contributors to these findings.

More than 150 articles have been published on urinary proteomics and the kidney over the last 10 years, a substantial proportion being review articles. Initial studies focused on acute kidney failure [15], transplantation rejection [12], renal carcinoma [11], obstructive nephropathy [16], and glomerulonephritis [17, 18]. Different biomarkers and panels of biomarkers have demonstrated sensitivities and specificities ranging 0.45–0.98. However, many studies have been based on small numbers, there has been a lack of relevant clinical information, and results have been difficult to reproduce due to difficulties and variations in analytical techniques and sample preparation. Furthermore, many studies have so far been carried out in settings with little clinical relevance, e.g. the patients had already been properly diagnosed with a simpler and cheaper test (s-creatinine, u-dipstick, ultrasound, etc.). Urinary proteomic tests have therefore not come into clinical practice.

However, more recently Good et al. studied chronic kidney disease with capillary electrophoresis coupled to mass spectrometry (CE-MS) [10]. They found a very high diagnostic accuracy (area-under-ROC-curve 0.955) when testing 379 healthy subjects versus 230 CKD patients (the majority having glomerulonephritis and diabetes nephropathy). CE-MS has emerged as a promising technique with stable and reproducible results over time and in different cohorts [17, 19–21]. Our study also find a similarly high diagnostic accuracy (AUC 0.977), and we extend the results to patients with hypertensive nephropathy. This is, as far as we know, the first report on urinary proteomics for diagnostics in the large and increasing group of patients with CKD caused by hypertensive nephropathy.

A central question is whether urinary proteomics can improve clinical handling of patients beyond what is possible with current diagnostic tests. CE-MS based urinary proteomics was recently found to have better ability to predict which patients with diabetes mellitus would progress to diabetes nephropathy over the next 5 years compared to microalbuminuria testing (areas under ROC curves 0.93 and 0.67, respectively) [22]. Using the same analytical methods, we found that urine proteomics testing in combination with albuminuria was able to classify rapid progressors versus slow progressors significantly better than albuminuria alone. We found that urine proteomics contributed important additional information, i.e. it increased the area under ROC curve at the magnitude of 0.15 beyond what was achieved with albuminuria. Typically even major risk factors like HDL cholesterol provide only marginal additional value when evaluated with ROC (delta AUC 0.01) [23]. Furthermore, a large proportion of the big group of slowly progressing patients having been assigned an intermediate risk of progression were reclassified to the low risk group. Such reduction of the number of false positive cases is important in a potential CKD screening setting. Although we had a few more false negative cases, a stronger increase of true positive cases was seen, and the reclassification lead to an overall improvement of benefit versus risk. Finally, previous studies have used a proteomics score of 0.323 as cut-off for diagnosing CKD [10], but our study showed that the risk for accelerated kidney function loss starts with scores above 0.0.

Excessive accumulation of extracellular matrix and subsequent fibrosis is a general pathophysiological mechanism involved in many if not all types of progressive kidney disease. If the triggering event is not cleared, epithelial tubular cells will transition to a more mesenchymal like cell type starting a chronic interstitial process with increased production of collagen type 1 and type III [24]. Both experimental and human studies have suggested an initial phase with increased extracellular matrix production followed by an imbalance between collagen degradation enzymes (matrix-metallo-proteinases, MMPs) and their tissue inhibitors (tissue inhibitors of metalloproteinases, TIMPs) leading to reduced degradation, favoring the development of tubulointerstitial fibrosis [24, 25]. Previous studies have found correlations between increased urine levels of procollagen III, which probably is a marker of increased production of collagen, and the extent of interstitial fibrosis [26]. This is not necessarily in opposition to our findings in the urine from rapid CKD progressors of reduced amounts of collagen types I–III fragments, which more likely is a marker of reduced collagen breakdown, rather than of increased collagen production. Reduced urinary collagen has been found consistently in CKD patients using the same CE-MS technology as in our study [10, 19–21]. Lower levels of urinary MMP activity has also been found in progressive compared to stable patients with diabetes nephropathy [27]. Potentially this could be useful for the development of future CKD biomarkers of rapid progression, and our data indicate that this could hold for hypertensive nephropathy patients as well. However, there has been conflicting reports on this complex topic [28], and influence of CKD-bone-mineral-disorder has also been proposed.

Several other urinary protein findings in our study also support on-going interstitial inflammation, fibrosis and tubular damage with similar results across CKD diagnosis, including hypertensive nephropathy. Uromodulin is exclusively produced in tubular cells in the thick ascending limb of Henle’s loop and increasingly found to be associated with kidney disease [29]. Low urine levels are associated with rapid progression of CKD and have been found in cases with tubular atrophy and fibrosis [30, 31] CD99 antigen is expressed in most tissues, including the kidney, and it is important for the ability of leucocytes to extravasate into the interstitium as part of the inflammatory process [32]. Low urine levels have been associated with kidney disease in other cohorts also, but the pathophysiological reason for this is unclear. Osteopontin, which is excreted when cells in the distal tubules are stressed [33], was also reduced in urine from CKD patients. Osteopontin is involved in remodelling of the extracellular matrix and inhibition of apoptosis [34]. Similar findings have been reported from IgA patients, while patients with membranous glomerulonephritis and minimal change nephropathy who typically have minor tubular and interstitial damage were reported to have normal urine levels [35]. Several of the proteins found in excess in our CKD urine samples are normally reabsorbed in the proximal tubules. Our findings are therefore in line with previous reports and strongly indicate the presence of interstitial and tubular damage in most types of CKD, including non-biopsy verified hypertensive nephropathy. Such patients have often been claimed to only have normal age related reduction of GFR, but at least when eGFR is below 30 ml/min/1.73 m^2^ they seem to be suffering from the common pathophysiological pathway found in most types of progressing CKD.

The current study has some limitations. First, the number of participants was rather low, which could lead to loss of precision and risk of type 2 errors. Second, we compared patients with rather advanced CKD stages with healthy subjects, so the differences between the groups were presumably large. Also, we measured albuminuria by dipstick, which is less precise than urinary albumin/creatinine ratio. However, the association of the dipstick test with both mortality, cardiovascular morbidity and CKD progression has been well validated in large international studies [36]. Also, modern dipsticks have a very high diagnostic accuracy for macroalbuminuria (area-under-ROC-curve 0.99) [37], which is often the range of interest for predicting progression rate in clinical practice. Also, the ideal measure of GFR decline would be strictly prospective, i.e. after inclusion. However, long observation time with several creatinine measurements provides a more robust measure of GFR decline, and additional sensitivity analysis based on GFR decline after baseline, albeit with fewer subjects and measurements due to deaths and start of renal replacement therapy, gave similar results (adding the urine proteomics score to dipstick testing increased the area under the ROC curve from 0.91 to 0.97). One of the strengths of this study is that it was based on a clinically relevant and well described patient cohort. The CE-MS method for urine proteomic analysis has high sensitivity and reproducibility [38]. It is also insensitive to interfering compounds and enables measurement of the relative abundance of the peptides using internal standards (i.e. semi-quantitative measurements with very low coefficients of variation combined with a large human urinary peptidome database enabling identification of significant changes in biomarkers).

In conclusion, a panel of urinary proteins was able to accurately diagnose CKD, and in combination with albuminuria was significantly better to detect patients with rapid kidney function decline than albuminuria alone. Reduced urine levels of collagen types I and III, uromodulin, CD99 antigen and osteopontin, all implicated in fibrosis-promoting processes such as extracellular matrix deposition, inflammation and reduced collagen breakdown, were found in our CKD patients. Urine proteomics performed equally well in patients with hypertensive nephropathy as in patients with other CKD causes. Further studies with prospective designs using this new potentially useful tool are highly required.

## Methods

We included consecutive CKD patients with eGFR below 30 ml/min/1.73 m^2^ not yet on renal replacement therapy (RRT), from the outpatient clinic at St Olav’s Hospital, Trondheim, Norway, in December 2009–February 2010. A convenient sample of healthy persons working in our department not taking any medication and with no history of CKD, cardiovascular disease or diabetes, was included as controls. Age, sex, CKD diagnosis, and blood pressure were recorded in all participants. Blood was drawn for standard evaluation of kidney function. A second morning urine sample was tested with a dipstick, and then immediately frozen to −20 and thereafter to −80°C within 24 h. Creatinine measurements available prior to inclusion and over a 2.5 years follow-up period were recorded in order to calculate eGFR decline per year using the CKD-EPI equation [39]. Individual linear regression analyses of eGFR decline were performed to compare kidney function decline before and after inclusion. Blood pressure was measured as the average of the last two out of three measurements at inclusion.

Urine samples were prepared as previously discussed [10]. Briefly, after dilution with urea, ammoniumhydroxyde, and sodium dodecyl sulfate, the 0.7-ml aliquots of urine were ultrafiltered in order to remove proteins of higher molecular mass (>20 kDa), desalted, lyophilized, and stored at −20°C. The samples were resuspended in HPLC grade water shortly before capillary electrophoresis/mass spectrometry analysis. CE-MS analysis was performed with a P/ACE MDQ capillary electrophoresis system (Beckman Coulter, Brea, CA, USA) coupled on line to a micro-TOF–MS instrument (Bruker Daltonics, Bremen, Germany) [40].

For data processing, mass spectral ion peaks representing identical molecules at different charge states were deconvoluted into single masses using Mosaiques Visu software [41]. For normalization of analytical and urine dilution variances, MS signal intensities were normalized relative to 29 internal standard peptides generally present in at least 90% of all urine samples with small relative standard deviation. For calibration, linear regression was performed. All detected peptides were deposited, matched, and annotated in a Microsoft SQL database (Microsoft, California).

Previous CE-MS measurements of urine samples have resulted in a maximum of 5,010 distinct peptides, which describes the human urinary low molecular-weight proteome [42]. The CKD273-classifier is a support vector machine (SVM)-based classification model [43–45], which allows the classification of samples in the high dimensional parameter space using MosaCluster software (version 1.7.0) [46]. Applying the CKD273-classifier to CE-MS data of unknown samples, MosaCluster calculated classification scores, based on the amplitudes of the 273 CKD biomarker peptides. Classification was performed by determining the Euclidian distance (defined as the SVM classification score) of the 273-dimensional vector to a 272-dimensional maximal margin hyperplane, which was defined previously [10]. The cut-off of the classification score was previously determined from the result of the biomarker discovery cohort in Good et al. [10]. Patients with urine samples who had classification scores exceeding 0.343 were classified as CKD273 classifier positive cases and patients with urine samples scoring below 0.343 were classified as CKD273-classifier controls [10]. Quantitative differences of individual proteins between cases (glomerulonephritis/diabetes nephropathy, hypertensive nephropathy, or other CKD diagnosis) and control subjects were calculated. Statistical significance was assumed at unadjusted p < 0.05 with the Wilcox test. All data were calibrated and annotated to the Mosaiques human urinary database [47].

Statistical analysis were done using Stata 13.1 software (StataCorp, TX, USA). Decline in kidney function over time was compared with linear regression analysis. Mean proteomic score in different groups were compared with two-sample t test. Stata function “roccomp” was used to test for ROC area equality of logistic regression based models (e.g. base model including albuminuria versus enhanced model including albuminuria plus proteomic score). We also used the Stata function “incrisk” from Longton and Pepe, which is a collection of performance improvement measures comparing a base model versus an enhanced model. Significance testing is difficult in risk reclassification, but these are useful for demonstrating how different risk prediction models changes the risk estimates in subjects with and without the outcome [48]. Predicted risk for rapid kidney function decline below 10% were defined as low, risk above 50% as high, and 10–50% as intermediate.

The study was approved by the Regional Committee for Medical and Research Ethics. It was carried out in accordance with the Declaration of Helsinki. All participants gave written consent.
